# The Many Faces of Philadelphia: A Mature T-Cell Lymphoma with Variant Philadelphia-Translocation and Duplication of the Philadelphia Chromosome

**DOI:** 10.3390/hematolrep17010001

**Published:** 2025-01-06

**Authors:** Livia Vida, Bálint Horváth, Miklós Egyed, Béla Kajtár, Hussain Alizadeh

**Affiliations:** 1 Department of Pathology, Medical School, University of Pécs, Szigeti Str. 12, 7624 Pécs, Hungary; vida.livia@pte.hu (L.V.);; 2 Department of Internal Medicine, Kaposi Mór Teaching Hospital of Somogy County, Tallián Gy. Str. 20-32, 7400 Kaposvár, Hungary; 3 Department of 1st Internal Medicine, Medical School, University of Pécs, Ifjúság Str. 13, 7624 Pécs, Hungary

**Keywords:** T-cell prolymphocytic leukemia, T-PLL, *BCR::ABL1*, Philadelphia translocation, e6a2, variant transcript

## Abstract

**Background:** T-cell prolymphocytic leukemia (T-PLL) is a rare mature T-cell lymphoma that is usually associated with poor prognosis and short overall survival. **Methods:** We present a case of a 61-year-old woman presenting with T-PLL and the leukemic cells harboring *BCR::ABL1* (*BCR*—breakpoint cluster region; *ABL1*—ABL protooncogene 1) fusion transcripts as the result of a variant of t(9;22)(q34;q11) called Philadelphia translocation: t(9;22;18)(q34;q11;q21). Sequencing revealed a rare *BCR* transcript with an exon 6 breakpoint corresponding to e6a2 transcripts, which has thus far been reported in only 26 cases of leukemias. **Results:** After 9 months of follow-up, the disease progressed and required treatment. Following alemtuzumab and chemotherapy, a short course of imatinib therapy stabilized the disease for six months, which was followed by progression and the demise of the patient. **Conclusions:** To the best of our knowledge, this is the first report of a mature T-cell lymphoma with a variant Philadelphia-translocation and a very rare type of *BCR::ABL1* transcript. This case highlights the importance of comprehensive genetic testing of malignancies, as abnormal molecular pathways may be uncovered that may be specifically targeted by drugs.

## 1. Introduction

The *BCR::ABL1* (*BCR*—breakpoint cluster region; *ABL1*—ABL protooncogene 1) translocation is one of the most extensively studied abnormalities in tumor cytogenetics, resulting in the iconic Philadelphia chromosome (Ph-chromosome), which is a shortened version of chromosome 22. This translocation is detectable in chronic myeloid leukemia (CML), a proportion of acute lymphoblastic leukemia (ALL), and very rarely in acute myeloid leukemia (AML) cases [1,2]. T-cell lineage involvement is very uncommon, and is restricted to uncommon cases of T-cell ALL and T-cell lymphoblastic crises of CML [3,4,5]. The *BCR::ABL1* translocation is not associated with mature lymphoid neoplasms, although a few such cases have been reported, with a single report involving mature T-cell lymphoma [6,7,8].

Although in CML patients the *BCR::ABL1* translocation is detectable not only in myeloid cells, but in hematopoietic stem cells and progenitors of both B and T-cells, it is not present in mature T-cells [9]. The explanation of this phenomenon is unclear. It seems that *BCR::ABL1*^+^ T-cell progenitors have a reduced differentiation capability and reduced survival [10].

Here, an exceptional case of *BCR::ABL1*-positive T-cell prolymphocytic leukemia case is reported. A very rare *BCR::ABL1* transcript variant was identified in the neoplastic cells, as well as a variant Ph-chromosome with evidence of *BCR::ABL1*-related clonal evolution. Bone marrow progenitors and myeloid cells were *BCR::ABL1*-negative, and the abnormality was restricted to the neoplastic T-cells. The patient received various treatments, and the disease was stabilized temporarily while the patient was receiving tyrosine kinase inhibitor therapy; however, the patient later died following progression after 39 months of survival. The objective of this manuscript is to expand the knowledge base of known cancer drivers occurring in unusual clinical contexts by providing a description of this unique T-cell lymphoma case.

## 2. Case Presentation

In May 2012, a 61-year-old female patient presented with complaints of weakness and itching for six weeks. Splenomegaly and mild leukocytosis (30 G/L) without lymphocytosis, normal platelet count and hemoglobin values were detected, no B symptoms were reported, and LDH (lactate dehydrogenase) was found to be elevated (607 U/L). A mild, generalized lymph node enlargement was demonstrated with imaging. Hepatosplenomegaly was not detected. Informed consent was given by the patient and the study was approved by the institutional review board (IRB), with reference number 6650/PTE-2017.

**Figure 1:**
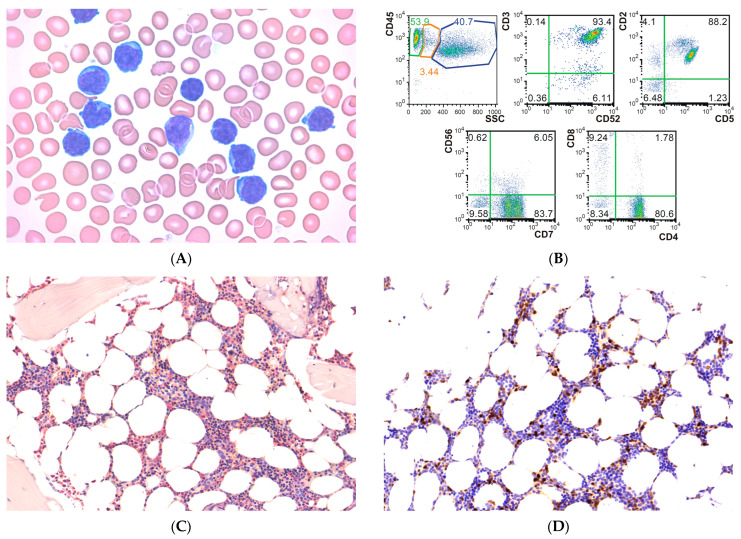
Morphology and immunophenotype of neoplastic cells in peripheral blood and bone marrow. (**A**) Peripheral smears revealed neoplastic lymphocytes of 9–10 µm, with irregular, often angulated nuclei and prominent nucleoli (1000× magnification). (**B**) Flow cytometry at diagnosis showed 54% lymphocytes, the majority of which demonstrated the following phenotypes: CD2^+^/CD3^+^/CD4^+^/CD5^+^/CD7^+^/CD8^−^/CD52^+^/CD56^−^. SSC indicates side scatter. Green gate: lymphocytes; orange gate: monocytes; blue gate: granulocytes. The numbers on the other scatter plots represent percentages of the quadrants. (**C**) Histology showed normocellular bone marrow with approximately 15% interstitial and vaguely nodular lymphoid infiltrate in the bone marrow (200× magnification, Naphthol AS-D chloracetate esterase staining. (**D**) Immunohistochemistry using TCL1 antibody (Abcam, Cambridge, UK, 1:100 dilution), 200× magnification.

Peripheral blood smears showed 46% of atypical lymphocytes with slightly angulated, 8–10 µm large nuclei and prominent nucleoli (Figure 1A). Immunophenotyping of peripheral blood and bone marrow aspirates was performed by flow cytometry using a Beckman-Coulter Navios flow cytometer. Peripheral blood and bone marrow showed 44% and 33% of CD2^+^, CD3^+^, CD4^+^, CD5^+^, CD7^+^, CD8^−^, CD52^+^, CD56^−^ T-cells, respectively (Figure 1B). Bone marrow biopsy showed 10% of focal, interstitial lymphoid infiltrate showing CD3, CD4 and TCL1 (T-cell leukemia/lymphoma protein 1, protein product of *TCL1A* gene) positivity, as well as TDT (terminal deoxynucleotidyltransferase), CD10, and CD34 negativity with normal hematopoiesis in the background (Figure 1C,D). Monoclonal *TCRG* (T-cell receptor gamma) gene rearrangement was demonstrated using polymerase chain reaction. Next-generation sequencing (NGS) was performed on Novaseq 6000 platform (Illunina, San Diego, CA, USA) using a customized panel of 138 genes involved in myeloid and lymphoid leukemias with a median sequencing depth of 1052X and coverage of 92.4% at 500X depth (Supplementary File S1). No pathogenic variant of the JAK/STAT pathway was detected.

**Figure 2:**
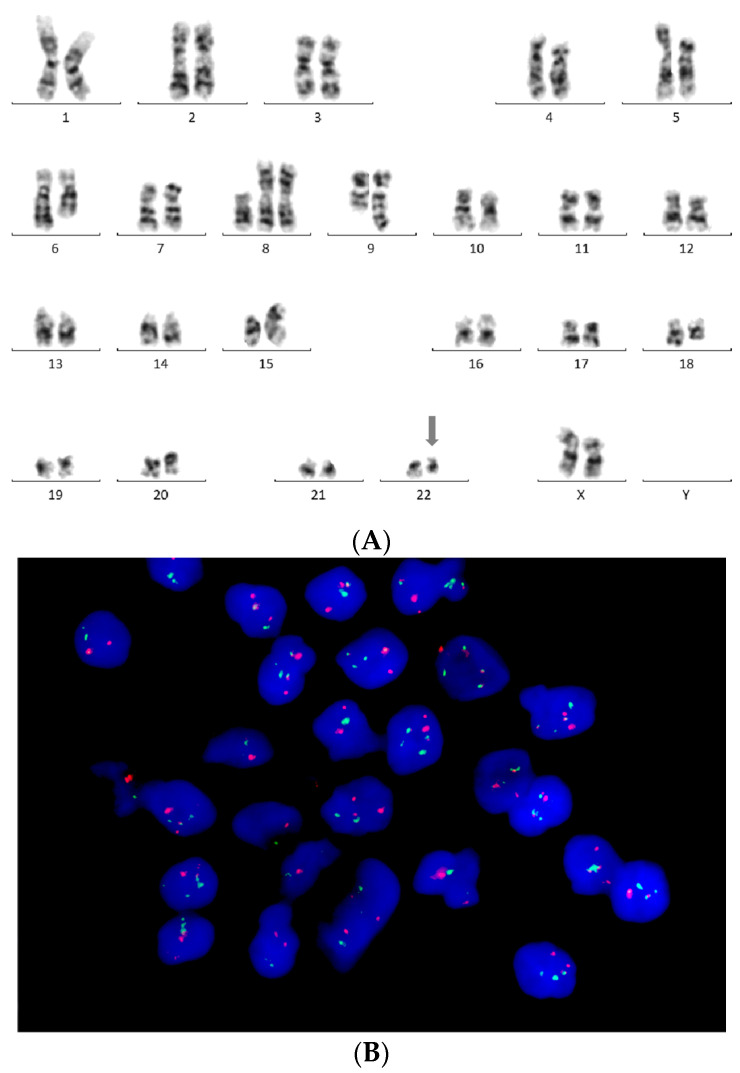
Cytogenetic findings. (**A**) 47,XX, del(6)(q22q23), i(8)(q10), +i(8)(q10), t(9;22;18)(q34;q11;q21), inv(14)(q11;q32), der(15)t(8;15)(q22;p13), add(16)(p13) karyotype was seen in 5 out of 30 metaphases obtained from peripheral blood (an arrow marks the Philadelphia chromosome). Four metaphases showed the same abnormalities with the addition of add(17)(q25), while one metaphase showed the addition of +der(22). (**B**) Interphase cells showed one fusion, two red, and two green signals with *BCR::ABL1* dual fusion probe (Abbott, Chicago, United States of America). Approximately half of the cells showed an additional fusion signal representing +der(22) (630×).

**Figure 3:**
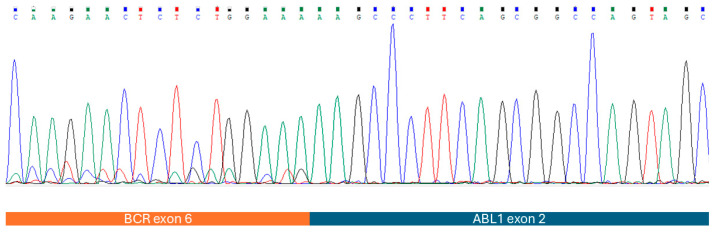
Results of Sanger sequencing. Based on alignment with reference RNA sequences, the transcript was identified as in-frame with the breakpoint showing fusion of *BCR* exon 6 to *ABL1* exon 2.

Karyotyping of peripheral blood cells stimulated by phytohemagglutinin for three days, and bone marrow karyotyping after 24 h of culturing and GTG banding revealed a complex karyotype with a typical inv(14)(q11;q32) along with del(6q), i(8q) and a variant Philadelphia translocation—t(9;22;18)(q34;q11;q12)—as well as duplication of the Philadelphia chromosome. All metaphases with clonal alterations (10/30 in peripheral blood and 11/18 in bone marrow) showed del(6q), i(8q), inv(14), as well as t(9;22;18) and two other structural aberrations (Figure 2A). Fluorescence in situ hybridization (FISH) was performed using *BCR::ABL1* dual fusion (Abbott-Vysis), as well as TCL1A break apart (Cytocell) probe kits. Fusion of *BCR* and *ABL1* genes (Figure 2B), as well as the disruption of the *TCL1A* gene, was demonstrated. Myeloid cells and megakaryocytes were found to be *BCR::ABL1*-negative based on FISH performed on bone marrow biopsy slides. Half of the leukemic cells showed a signal pattern indicating duplication of the Philadelphia chromosome. Following reverse-transcriptase polymerase chain reaction (PCR) using primers for *BCR* exon 1 and *ABL1* exon 2, Sanger sequencing of the transcript revealed an e6a2 *BCR::ABL1* transcript indicating an atypical *BCR* breakpoint (Figure 3). The cytogenetic and molecular findings remained unchanged during follow-up.

**Figure 4:**
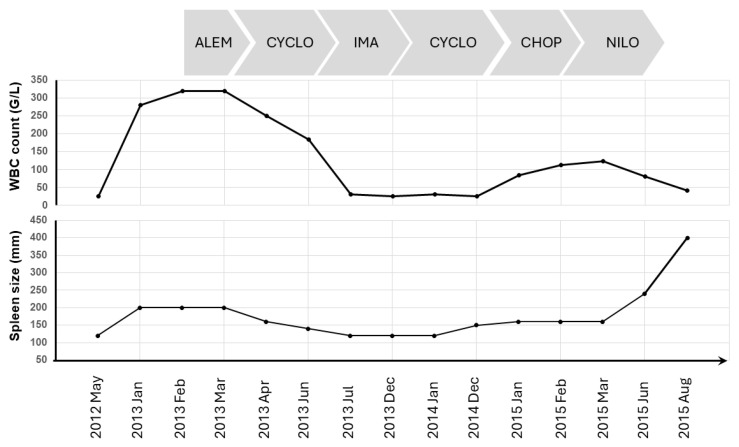
Summary of therapies and clinical data. ALEM: alemtuzumab; CYCLO: cyclophosphamide; IMA: imatinib; CHOP: cyclophosphamide-, doxorubicin-, vincristine-, and prednisone-containing regimen; NILO: nilotinib.

Initially, therapy was not started for 9 months due to minimal progression. Then, alemtuzumab therapy was initiated due to significant leukocytosis, night sweats, fever and mild splenomegaly, but no response was observed for 2 months. Cyclophosphamide therapy was started at a dose of 100 mg daily, and a reduction in leukocyte count as well as spleen size was achieved. Three months later, imatinib was initiated at a 400 mg dose. Both leukocyte count and spleen size improved; however, therapy was stopped due to the appearance of skin rashes six months later. No progression was noted during this time. Cyclophosphamide therapy was reinitiated. Twelve months later, a multiagent regimen containing cyclophosphamide, doxorubicin, vincristine and prednisone (CHOP) was started due to an increase in the patient’s spleen size and white blood cell count. Two months later, nilotinib was initiated due to progressive disease, but no significant clinical improvement was seen for four months. Eventually, a massive splenomegaly occurred, and the patient was lost 39 months after the initial diagnosis. Figure 4 summarizes the sequence of therapies (Figure 4).

## 3. Discussion and Conclusions

The *BCR::ABL1* translocation and the resulting fusion protein is an archetype of molecular pathological abnormalities that may be successfully targeted with specific drugs. *BCR::ABL1* signaling results in increased survival and proliferation of hematopoietic progenitors, as seen in cases of CML, a proportion of ALL, and rarely in AML [1]. The *BCR::ABL1* translocation is not associated with other cancer types, although *BCR:ABL1* positivity was reported in three plasma cell myeloma cases [11,12,13], two diffuse large B-cell lymphomas [14,15], one hairy cell leukemia [6], and a single case of Sezáry syndrome transforming into large T-cell lymphoma [7].

The case reported herein is unique for several reasons. It is the first reported case of Philadelphia translocation-positive T-cell prolymphocytic leukemia. T-PLL is a rare, mature, post-thymic T-cell lymphoma with a poor prognosis and short overall survival [16]. The median age at diagnosis is 61 years. Although the clinical course is usually aggressive, with very high WBC count and splenomegaly from the onset of disease, approximately 15% of patients show an indolent phase of variable length, during which patients may be asymptomatic [16,17]. T-PLL cells are characteristically CD2^+^/CD3^+^/CD5^+^/CD7^+^/CD52^+^, and 60% of cases show CD4 positivity and CD8 negativity, although co-expression of CD4 and CD8, and rarely, CD4^−^/CD8^+^ phenotypes is observed [18]. Additionally, 75% of cases show structural abnormalities of chromosome 14 involving the gene *TCL1A*. Inversion 14 is the most common of these abnormalities [12]. Trisomy or isochromosome 8 is seen in approximately 66% of cases [19].

In addition to the aforementioned characteristics, the neoplastic cells of the reported case showed duplication of the Philadelphia chromosome, a common additional cytogenetic abnormality seen associated with clonal evolution in cases of CML. This aberration indicates the importance of *BCR::ABL1* signaling regarding survival and proliferation of the neoplastic T-cells, since the duplication may increase the level of *BCR::ABL1* expression and is regarded as a potential cause of resistance to tyrosine kinase inhibitors [1,20].

The *BCR::ABL1* fusion gene was the result of a variant Philadelphia translocation involving chromosomes 9, 18, and 22. Such variant translocations are observed in approximately 5–10% of CML cases. Molecular studies revealed a rare *BCR::ABL1* transcript (e6a2). This transcript has been reported in less than 30 cases to date; 18 CML, 1 ALL, and 7 AML cases [21,22,23]. It has been suggested that this transcript may be associated with more aggressive clinical behavior in CML [22,24]. The limited number of reported cases does not allow for definitive conclusions to be drawn; however, four out seven patients reported to have received imatinib treatment and follow-up experienced treatment failure.

The patient received only a short course of tyrosine kinase inhibitor (TKI) therapy. Although a complete response was not observed, the patient’s disease stabilized; however, it was not possible to draw conclusions regarding the TKI sensitivity of the neoplastic T-cells. T-PLL is characterized by *TCL1A* fusion, usually in the form of inv(14), as was detected in the case discussed here. Amplification of 8q is a common secondary finding; complex karyotypes are detected in >70% of cases [25]. Mutations of the JAK/STAT pathway are common alterations which occur in 76% of cases, with *JAK3* mutations being most common [26]; however, activation of the Jak/Stat pathway may be detected even without gain-of-function mutations [27]. *ATM* deletions and mutations, as well as mutations of methyl transferases affecting epigenetic modulation (e.g., *KMT2C*, *KMT2D*, *KMT5A*), are also common [28].

Our case is unique, being the first and, presently, only reported incidence of *BCR::ABL1*-positive T-PLL. A single case of T-PLL with *SEPT9::ABL1* fusion has been reported previously. Other cytogenetic or molecular alterations were not reported in that case, and the patient did not respond to imatinib or dasatinib [29].

T-PLL is a rare, mature T-cell lymphoma with an aggressive clinical course. Typically, patients present with leukocytosis, splenomegaly, and lymphadenopathy, often with anemia and thrombocytopenia. Initially, an indolent period may be detected; however, progression does occur, and the median overall survival is less than 3 years. The current recommended first-line therapy is alemtuzumab, which should be followed by hemopoietic stem cell transplantation if possible. There is no current consensus regarding the best treatment after alemtuzumab failure, which occurs almost universally following a median of 12 months. Purine analogues as monotherapy or in combination are generally recommended in cases of T-PLL [30].

Recent genomic profiling results have led to preclinical studies suggesting that epigenetic modulation by histone deacetylase inhibitors may be a potential therapeutic approach in T-PLL [30]. Antagonists of Mdm2 or Bcl2 family molecules as well as inhibitors of the Jak/Stat pathway show promise in vitro, with few clinical case reports or short case series available [31,32,33,34].

It is not yet clear how *BCR::ABL1* affects the survival and proliferation of cells other than stem cells or progenitor cells. The *BCR::ABL1* fusion results in constitutive activation of the Jak/Stat pathway, often due to direct phosphorylation of STAT5 [35,36], which may lead to activation of this crucial pathway even without other gain-of-function mutations, The fusion may result in increased proliferation or resistance to apoptosis of cells by activating other signal transduction pathways such as RAS/RAF/MAPK, WNT or PI3K/AKT/mTOR [37]. It is currently unclear why *BCR::ABL1* fusion is rare outside the context of ALL and CML despite influencing several crucial signaling pathways. It is possible that genomic alterations may not maintain the same biomarker potential across different diagnoses. Investigating how *BCR::ABL1* or other known driver mutations influence signal transduction pathways in unusual diagnoses may lead to a better understanding of the mechanics of targeted therapy, and even to more efficient control of minimal residual disease in *BCR::ABL1*-positive leukemias. Also, unexpected targetable genetic alterations appearing in T-cell malignancies may provide therapeutic as well as research opportunities, potentially leading to better understanding and thus, better control of these often difficult-to-treat malignancies.

In summary, the case reported here is, thus far, the only *BCR::ABL1*-positive T-PLL case recorded in the literature. The fusion transcript was rare, corresponding to e6a2 transcripts that have been reported in only 26 cases so far. The findings suggest that *BCR::ABL1* fusion was a driver mutation in this case, as no other genetic alteration was noted to activate the Jak/Stat pathway. A single, unique case does not provide sufficient information to draw conclusions regarding the exact biological consequence of *BCR::ABL1* fusion in mature T-cell lymphomas and the efficacy of targeted therapy in this context. Appropriate models have to be designed to assess the exact molecular biological role of *BCR::ABL1* in the context of mature T-cell neoplasms. Our case demonstrates the crucial importance of karyotyping and comprehensive genomic testing of leukemias, since potential targets of efficient therapies may be uncovered. Continuous expansion of our knowledge base of genetic alterations in cancer is vital for increasing the efficiency of therapy.

## Data Availability

Dataset available on request from the authors.

## Supplementary Materials

The following supporting information can be downloaded at https://www.mdpi.com/article/10.3390/hematolrep17010001/s1, Supplementary File S1: List of next-generation sequencing genes used in the manuscript.
