# Supplementary material for: The Many Faces of Philadelphia: A Mature T-Cell Lymphoma with Variant Philadelphia-Translocation and Duplication of the Philadelphia Chromosome
Source: Hematol Rep. 2025 Jan 6;17(1):1. doi: 10.3390/hematolrep17010001 (PMC11755446; doi:10.3390/hematolrep17010001)
Supplement: Supplementary file 1 [file hematolrep-17-00001-s001.zip › hematolrep-3302215-supplementary.pdf]

Gene list of the custom panel used for next-generation sequencing:

ABL1, ABL2, ACD, AKT1, AKT2, ANKRD26, ARID1A, ASMTL, ASXL1, ASXL2, ASXL3, ATRX, BCL11B, BCL2, BCL2A1, BCL2L2, BCL6B, BCL9, BCL9L, BCOR, BCORL1, BRAF, BTK, CALR, CBL, CCND3, CDKN2A, CDKN2B, CEBPA, CNOT3, CREBBP, CRLF2, CTCF, CUX1, CSF3R, DDX3X, DDX41, DNM2, DNMT1, DNMT3A, DOT1L, EBF1, EP300, ERCC6L2, ERCC6L2, ETNK1, ETV6, EZH2, FBXW7, FLT3, GATA1, GATA2, GATA3, GNAO1, GNAS, GNB1, IDH1, IDH2, IKZF1, IKZF2, IKZF3, IL7R, JAK1, JAK2, JAK3, KDM6A, KIT, KMT2A, KMT2C, KMT2D, KRAS, LEF1, MPL, MSH6, MYC, NCOR1, NF1, NOTCH1, NOTCH2, NOTCH3, NPM1, NR3C1, NRAS, NT5C2, NTRK3, PAX5, PDGFRA, PHF6, PIK3CD, PIK3R1, PRPF8, PRPS1, PTEN, PTPN11, RAD21, RB1, RPL10, RPL22, RUNX1, SAMD9, SAMD9L, SETBP1, SETD2, SF3B1, SH2B3, SMARCA4, SMARCC2, SMC1A, SMC3, SRP72, SRSF2, STAG2, STAT1, STAT2, STAT3, STAT5A, STAT5B, TBL1XR1, TCF3, TERC, TERT, TET2, TINF2, TP53, TSC1, TUSC3, TYK2, U2AF1, UBA2, USH2A, USP7, USP9X, VPREB1, WHSC1, WT1, ZEB2, ZFP36L2, ZRSR2
